# Low-Power Wearable Respiratory Sound Sensing

**DOI:** 10.3390/s140406535

**Published:** 2014-04-09

**Authors:** Dinko Oletic, Bruno Arsenali, Vedran Bilas

**Affiliations:** Faculty of Electrical Engineering and Computing, University of Zagreb, Unska 3, 10000 Zagreb, Croatia; E-Mails: dinko.oletic@fer.hr (D.O.); barsenal@umcutrecht.nl (B.A.)

**Keywords:** wearable sensor, respiratory sounds, wheeze detection, short-term Fourier transform, decision trees, DSP, low-power implementation

## Abstract

Building upon the findings from the field of automated recognition of respiratory sound patterns, we propose a wearable wireless sensor implementing on-board respiratory sound acquisition and classification, to enable continuous monitoring of symptoms, such as asthmatic wheezing. Low-power consumption of such a sensor is required in order to achieve long autonomy. Considering that the power consumption of its radio is kept minimal if transmitting only upon (rare) occurrences of wheezing, we focus on optimizing the power consumption of the digital signal processor (DSP). Based on a comprehensive review of asthmatic wheeze detection algorithms, we analyze the computational complexity of common features drawn from short-time Fourier transform (STFT) and decision tree classification. Four algorithms were implemented on a low-power TMS320C5505 DSP. Their classification accuracies were evaluated on a dataset of prerecorded respiratory sounds in two operating scenarios of different detection fidelities. The execution times of all algorithms were measured. The best classification accuracy of over 92%, while occupying only 2.6% of the DSP's processing time, is obtained for the algorithm featuring the time-frequency tracking of shapes of crests originating from wheezing, with spectral features modeled using energy.

## 1. Introduction

Asthma is one of the most common chronic diseases, affecting more than 300 million patients worldwide. Long-term disease management is required in order to maintain the life quality of asthmatic patients and to prevent the progression of the disease. Management mainly consists of adherence to a prescribed medication plan and avoidance of asthmatic attack triggers. The occurrence of symptoms, such as “asthmatic wheezing” in respiratory sound, indicates a low level of control over the chronic disease [1].

Recently, medical devices for the quantification of wheezing appeared on the market [2]. The devices, operating on-demand in handheld form and operating overnight in holter form, were found to be useful in clinical trials for the diagnosis of asthma in children during bronchial challenge tests [3], for the monitoring of the response to therapy [4] and for the diagnosing of nocturnal asthma [5]. Nevertheless, the current practice of long-term asthma management still lacks a low-cost and wearable sensing system to empower patients and caregivers to continuously track the intensity of symptoms on their own. Recently, the advancement of low-power electronic technologies and the advent of smartphones enabled the design of sensing systems consisting of unobtrusive wearable sensors, measuring physiological signals, and a smartphone, serving as a gateway and interface for feedback to the patient [6–9].

**Figure 1.**
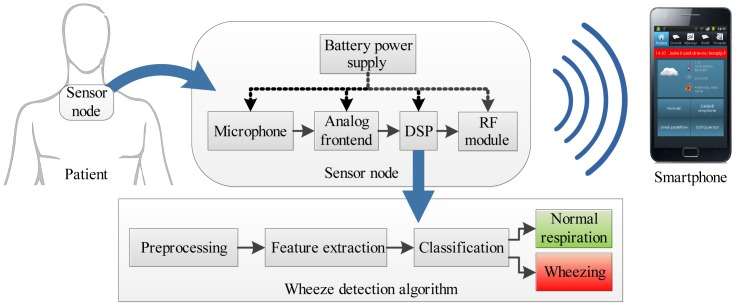
The concept of the system for the long-term acoustic monitoring of asthmatic symptoms. Abbreviations: DSP, digital signal processor; RF, radio-frequency module.

The concept of such a sensing system for the detection of asthmatic wheezing is shown in Figure 1. The battery-powered sensor node is worn on the skin surface. It consists of an acoustic sensor (microphone or accelerometer), a signal conditioning and an analog to digital conversion circuit (ADC), a digital signal processor (DSP) and a radio module communicating with the smartphone. A respiratory sound analysis algorithm performing real-time detection of wheezing is executed on the DSP on-board sensor node.

The low power consumption of the wearable, size-constrained wheeze detection sensor node is required in order to achieve long autonomy. In [10], the power consumption of such a sensor node was profiled, identifying the DSP and the radio module as the main consumers. The context of the medical application and the use of a smartphone as a peer device narrow the choice of radio modules to IEEE-802.15.1 (Bluetooth) and IEEE-802.15.4 (ZigBee) compliant, setting the boundaries of the radio power consumption [11]. Considering that the radio power consumption is kept minimal if transmitting only upon (rare) occurrences of wheezing, we focus on optimizing the power consumption of the DSP.

The main hardware prerequisites for low DSP power consumption are: (a) architectural features enabling efficient code execution (the number of instructions per clock cycle); (b) the high ratio of the processing speed with respect to the power consumed in the active state (millions of instructions per milliwatt); and (c) low power consumption in non-active states (standby, sleep, *etc*.) [10]. Following these guidelines, a Texas Instruments TMS320C5505 [12] 16-bit fixed-point audio/speech processor, featuring fast Fourier transform (FFT) unit, was chosen for this study.

In software, the DSP power can be lowered by minimizing portions of the time spent in the active state, by shortening the wheeze detection algorithm's execution time. Wheeze detection is performed on features obtained from time-frequency decompositions of respiratory sound: short-time Fourier transform (STFT), cepstral analysis, wavelets or linear prediction [13–16]. Most numerous are the algorithms using computationally fast STFT [17–23]. However, to the best of our knowledge, no work has been done regarding their mutual comparison in terms of the relation between their classification accuracies and execution speeds.

Thus, the contributions of this article are: (a) the review of STFT-based wheeze detection algorithms; (b) the analysis of the *a priori* computational complexity of the representative algorithms and their DSP implementation; (c) the test environment for the automated assessment of the classification accuracies of the algorithms running on the DSP; and (d) the analysis of the relation between the accuracies and execution times, for two scenarios of different detection fidelities: (1) the detection of the occurrence (event) of wheezing; and (2) the tracking of the wheeze duration.

The outline of the article is as follows: Section 2 describes the properties and the acquisition of the respiratory sound signal. Section 3 reviews the previous work on the detection of wheezing, focusing on STFT-based algorithms. Section 4 describes the implemented algorithms. Section 5 describes the evaluation methodology: the hardware platform, test signals, testing procedures and metrics. The results are listed in Section 6 and discussed in Section 7, and conclusions are drawn in Section 8.

## 2. Respiratory Sound Signal

### 2.1. Acquisition of Respiratory Sounds

Air streaming through airways produces mechanical vibrations, which are conducted through body tissues to the skin surface [24]. The human body's transfer characteristic is a low-pass-type with the parameters varying with the local tissue. On the skin surface, vibrations are sensed by a transducer, most commonly an electret-condenser microphones. The microphone is coupled to the skin surface through an air cavity formed by a shallow conical or bell-shaped enclosure attached to the skin. As an alternative, accelerometers can be attached directly to the skin surface [25]. Both the frequency characteristic and dynamics of the signal acquired at the output of the transducer are patient dependent and affected by: the measurement location [25], body posture [26], the geometry of the transducer coupling [27] and the transducer design [28].

Usually, the transducer output signal contains heart sounds concentrated below 60 Hz superimposed on the respiratory sound signal [29]. Thus, an analog bandpass filter is commonly used to isolate the respiratory sound signal band. An amplifier with a gain of 40–60 dB is required to adjust the dynamics of the microphone output (order of magnitude: 1–10 mV) to the input range of an analog to digital converter (ADC). Usually, the signal is digitized to 16-bit resolution, with a sampling frequency higher than 5,000 Hz [30].

### 2.2. Time-Frequency Properties of Respiratory Sounds

Normal respiratory sounds are cyclostationary, exhibiting the repetition of respiratory cycles. Each respiratory cycle can be divided into the inspiratory phase, the expiratory phase and the inter-respiratory pause. The respiratory sounds of the inspiratory phase usually exhibit a higher amplitude and are of a longer duration than the sounds during the expiratory phase [31]. Normal respiratory sounds' frequency spectrum is similar to a band-limited colored noise. The majority of the energy of the respiratory sounds acquired over lungs is typically grouped into the 100 to 250 Hz band, while tracheal sounds have a wider frequency band, with components extending to about 1,000 Hz.

Asthmatic wheezing is a time-continuous, tonal adventitious sound occurring during a fraction of the respiratory phase (either inspirium, expirium or both). It can last from tens of milliseconds to several seconds. Wheezing can be modeled as a single- or multi-component harmonic signal superimposed on the frequency spectrum of a normal respiratory sound. The harmonic components originating from wheezing typically appear in the frequency range between 100 and 1,500 Hz [31]. Both the amplitudes and instantaneous frequencies of the harmonic components of wheezing gradually change throughout its duration. In the rest of the text, we assume that the signal is divided into segments, short-enough in order to be considered stationary segment-wise, allowing us to track the temporally-evolving frequency content of respiratory sounds by STFT.

**Figure 2.**
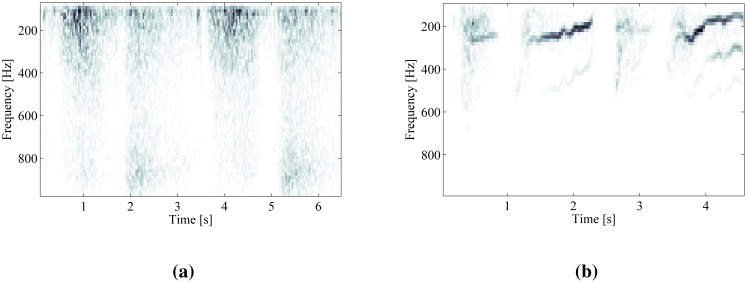
Time-frequency decomposition of respiratory sounds by short-time Fourier transform (STFT). (**a**) Two respiratory cycles of normal breathing; (**b**) two respiratory cycles containing wheezing.

A comparison of STFT time-frequency decompositions of normal respiratory sounds and respiratory sounds containing wheezing is shown in Figure 2. The harmonic components originating from wheezing appear as continuous frequency peaks elevated against the noise of normal respiration. The peaks of wheezing are localized along the frequency axis and spread in the direction of time axis.

## 3. Review of the STFT-Based Wheeze Detection Algorithms

This section provides an overview of the wheeze detection algorithms based on STFT decomposition. The employed preprocessing steps, feature extraction and classification methods are discussed. Table 1 summarizes the publications reviewed.

### 3.1. Signal Decomposition by STFT

The first step of a wheeze detection algorithm is the time-frequency decomposition of the respiratory sound signal in order to obtain its time-varying frequency content. Discrete STFT is used, because of the fast execution, despite its known limitations regarding temporal-frequency uncertainty.

Discrete STFT *X*[*m*, *k*] defined in Equation (1) calculates the *k*-point discrete Fourier transform of discrete-time windows *w*, sliding by step *m* over the signal, *x*. The non-rectangular window function is used to prevent spectral leakage due to finite window length *N*. Commonly, cosine window functions, such as Hann's or Hamming windows, are used. Furthermore, the overlap between successive windows may be used to show transients of a short duration in the signal [15].

(1) $$X\left[m,k\right]=\sum_{n=-\infty }^{\infty }x\left[n\right]w\left[n-m\right]e^{-j2\pi nk/N}$$

Most of the wheeze detection algorithms operate on power spectrum *P*[*m*,*k*] (Equation (2)) or amplitude spectrum *A*[*m*,*k*] (Equation (3)). In comparison to the amplitude spectrum, the power spectrum causes the attenuation of lower magnitude frequency components potentially containing the high-frequency harmonics of wheezing, due to the omission of the square root. Information from phase spectrum Φ[*m*, *k*] (Equation (4)) may also be used.

(2) P[m,k]=|X[m,k]|2=Re(X[m,k])2+Im(X[m,k])2

(3) $$A\left[m,k\right]=\sqrt{{\left|X\left[m,k\right]\right|}^{2}}$$

(4) Φ[m,k]=arctgIm(X[m,k])Re(X[m,k])

### 3.2. Preprocessing of the Spectrum

Preprocessing may include the following steps: equalization of the amplitude (or power) spectrum, spectral denoising and enhancement of the frequency resolution.

Equalization of the spectrum is performed for the compensation of individual patient and measurement site variations. The equalization step is implemented by detrending the spectrum of normal respiratory sound, thus leaving only high-magnitude spectral peaks standing out. Depending on later processing steps, it may be accompanied by the normalization of the spectrum in order to make it independent of respiratory flow. Early wheeze detection algorithms implemented equalization by subtracting the mean value from the power spectrum and, afterwards, normalization by dividing the spectrum by the standard deviation [32]. The equalization step was refined in [17] by dividing the spectrum into equidistant bands and performing band-wise detrending by the mean, followed by normalization using the band-wise standard deviation. In [19], the authors implemented equalization by point-wise detrending using a moving average filter.

Spectral denoising is used in order to reduce the number of isolated transient peaks (potentially producing false positives), but preserving spectral crests originating from wheezing. Wavelet denoising was proposed for this task by [20]. Some authors applied 2D image processing tools, such as bilateral edge preserving filtering [21] and Laplacian edge enhancing filtering [22], in order to enhance wheezes against the background noise in the spectrograms.

Enhancement of the frequency resolution of the STFT improves the frequency-localization of the spectral peaks originating from wheezing. Zero padding is the most straight-forward approach to increasing the frequency resolution [19]. Spectrogram reassignment and temporal-spectral dominance techniques of enhancement of the STFT time-frequency resolution were compared in [23].

### 3.3. Feature Extraction

Wheezing is discriminated from normal respiratory sound using spectral and temporal features extracted from STFT. The most commonly used are the features describing the shapes of the wheezing peaks in the time-frequency plane. Most algorithms using such features operate segment-wise, iterating two steps: (a) an extraction of spectral features (frequencies, the number of wheezing peaks, *etc*.) from the current signal segment, followed by; (b) tracking the temporal features (continuity, duration, *etc*.) using information from prior segments. In order to reduce the number of temporal features processed in Step (b), several approaches are proposed in Step (a) for the discrimination of the spectral shapes originating from wheezing, from the isolated peaks of the noisy respiratory spectrum.

Due to signal windowing, the discrete frequencies of wheezing are smeared across a band occupying several frequency bins in the amplitude (or power) spectrum, appearing as flattened “spectral crests”, rather than isolated discrete spectral peaks. A common approach of modeling the shape of such spectral crests is by low order statistical moments: the mean and variance (or standard deviation). This approach was first introduced in [32] by posing a set of relations between the mean value of different subsets of neighbor frequency bins surrounding each spectral maximum and the standard deviation of the whole spectrum. It was further refined by [17,20]. Both authors noticed that, if the spectrum has already been normalized (by the standard deviation) in the preprocessing step, the independence of the classification results from respiratory flow can be achieved by excluding the standard deviation from the spectral crest model. A different means of achieving flow independence was shown by [19]. There, due to the absence of the spectrum normalization step from preprocessing, the features describing spectral crests included both the mean and standard deviation, calculated locally around spectral maximums.

An alternative model of spectral crests was proposed in [18] with the aim of detecting only the audible sounds of wheezing. The audibility of a tonal signal masked in the noise of normal respiration was modeled by the ratio of the energy contained in the spectral crest to the energy contained in the noise of the normal respiratory sound. The bandwidth of such a wheezing crest was considered frequency-dependent, as analytically described by the psychoacoustic model.

The extraction of the spectral and temporal features describing wheezing crest shapes can also be performed simultaneously in time and frequency (on 2D spectrograms) by using image processing techniques. In [34], the detection of time-frequency plane crests was performed by gradient filtering. In [21], features describing centroid frequencies and the duration of spectral crests were calculated using edge detection Prewitt filtering, image closing and opening steps.

Apart from the features related to wheezing crest shapes, a variety of alternative STFT features were proposed in recent publications. One of the commonly used features is entropy, measuring the degree of grouping (clustering) of spectral components. Several variations are proposed. The difference and ratio between Shannon's entropy of probability mass functions of power-spectra maximums in successive time-windows were evaluated for single-feature classification in [37,40]. The mean distortion among sub-band histograms and the mean histograms of the sample entropy was evaluated in [36]. Rényi entropy was proposed in [38] as a measure of the time-domain signal's distribution uniformity. In addition, [38] evaluates the statistical parameters of kurtosis and the *f*_50_/*f*_90_ ratio as spectral features. These features were later compared in [39] to the spectral features describing signal tonality: spectral flatness and tonal index. This work has been extended in [41] in the direction of selecting the most discriminating feature set for wheeze detection by applying the minimal redundancy, maximal relevance technique, affirming the potency of spectral tonality. Of the other features, the cross-correlation index of successive spectra was proposed in [35]. Furthermore, an integral of time-varying power spectral content was used as a feature in [22].

### 3.4. Classification

Decision tree classifiers have most commonly been used in algorithms using features describing (tracking) the shapes of wheezing crests [17–20]. The tree structure is designed to track features describing spectral crests originating from wheezing in the time and frequency plane.

By employing a precise formalism, a linear support vector machine (SVM) classifier was used with wheezing crest shape features in [23]. A SVM was also utilized in [39] with spectral features describing tonality, spectral flatness, *f*_50_/*f*_90_, kurtosis and entropy.

Some authors used features derived from STFT as an input to a neural network (NN). The initial study of [33] investigated the usage of all STFT amplitude spectrum samples directly as NN input coefficients, identifying the need for input vector dimensionality reduction. This was addressed in [22] by using the projection of the spectrogram to frequency axis as features (NN). In a comprehensive study [15], neural networks were compared to vector quantization (VQ) and Gaussian mixture model (GMM) classification systems, with the average magnitudes of power spectral bands as features.

### 3.5. Review Summary

Table 1 summarizes the review of wheeze detection algorithms. Representative algorithms can be grouped into two groups. The first group is comprised of algorithms using features describing the shapes of wheezing crests, and the second group contains algorithms performing classification on alternative features. Several difficulties arise when comparing the results reported by different authors. First, it is unclear whether the features, other than those directly describing wheezing crest shapes, can provide sufficient information for accurate classification. Secondly, a variety of different datasets is used among the authors, as no publicly available standard dataset exist, containing normal and pathological respiratory sounds. Thirdly, classification accuracy testing methodologies and the associated accuracy reporting metrics vary. Nevertheless, two operating scenarios are commonly referred to: (1) the detection of the occurrence of sequences of wheezing; or (2) a wheezing sequence duration quantification. Finally, the execution speed of the proposed algorithms is seldom analyzed and reported.

## 4. Analysis of Implemented Algorithms

Following the presented review, we compare wheeze detection implemented using four algorithms, offering different levels of detection fidelity. The first two are the spectral crest shape tracking algorithms. The assumption is that such algorithms may provide the highest fidelity of wheeze classification, including estimation of the durations, number and frequency of the individual harmonic components composing the sound of wheezing. The algorithms differ by their spectral features: the first algorithm models the spectral crests using low-order statistical moments (mean and variance), building upon [17,19,20], and the second using energy (inspired by [18]).

The third algorithm also enables the estimation of the duration of wheezing, but does not enable distinguishing between individual frequency components. We implement the algorithm, tracking the duration of tonal intervals within the respiratory signal, facilitating a tonality feature recently proposed by [39].

The lowest fidelity algorithm is aimed solely at the detection of the occurrence of wheezing, without any prospect of estimating the duration of wheezing. We implemented the most representative of such algorithms, the one using Shannon's entropy of spectral peaks (as in [37]) to detect uniformity in the spectrum.

**Figure 3.**
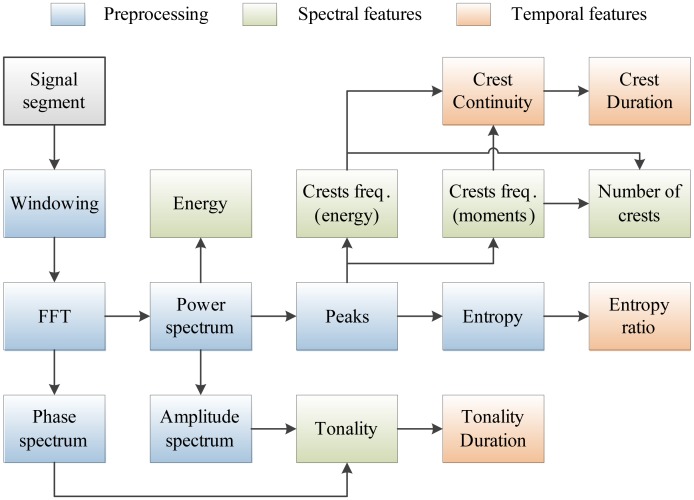
Program blocks used for features extraction from the respiratory sounds.

The complete set of features used in our work is shown in Figure 3. Features denoted as spectral are related to individual signal segments, while those denoted as temporal describe wheezing along the temporal axis in the time-frequency plane. The following sections describe the implementation of each program block and analyze their *a priori* computational complexity.

The analysis of computational complexity is performed by estimating the worst-case number of multiplications and additions, including multiplicative constants (additive constants are omitted from the analysis). No assumptions are made regarding any architectural specifics of the target DSP. Common elementary mathematical functions, listed in Table 2, are assumed to be implemented using the approximation methods listed in the column “Implementation”. Approximation methods are chosen to match the ones used in the experimental DSP implementation [42]. Their computational complexity, described by the associated variables, *N_itNR_*, *N_itN_*, *N_itTL_*, *N_itTS_*, *N_itTC_*, *N_itTA_*, defining their numerical precisions, is used throughout the analysis.

### 4.1. STFT Decomposition and Preprocessing of the Spectrum

Firstly, signal segments are windowed using the Hamming's cosine windowing function, and STFT is calculated according to Equation (1). Depending on the features to be extracted, STFT is followed by one or several of the following preprocessing steps. The power spectrum of the signal segment is calculated as in Equation (2). From the power spectrum, the amplitude spectrum (module) of the current signal segment is derived according to Equation (3). The phase spectrum is calculated according to Equation (4). The estimates of the *a priori* computational complexity of the signal decomposition and preprocessing program blocks are shown in Table 3.

### 4.2. Feature Extraction

#### 4.2.1. Signal Segment Energy

The energy of current signal segment *E*[*m*], defined in Equation (5), is used as the feature for the identification of respiratory pauses. The energy is calculated by the summation of the power spectrum components of the current segment.

Minimal and maximal energies *E_min_* and *E_max_*, given in Equation (6), are used as thresholds. They are obtained from the stored history of the previous segments' energies. The number of stored segment energies, *M_E_*, is chosen to cover the time-interval of at least one respiratory cycle.

(5) $$E\left[m\right]=\sum_{k}P\left[m,k\right]$$

(6) $$\begin{array}{cc} E_{\text{min}}=\text{min}\left(E\left[m-M_{E}\right]\ldots E\left[m\right]\right), & E_{\text{max}}=\text{max}\left(E\left[m-M_{E}\right]\ldots E\left[m\right]\right) \end{array}$$

#### 4.2.2. Spectral Tonality

Spectrum tonality is a feature describing the existence of the harmonic content within each signal segment. It is calculated as proposed in [40]. Firstly, the amplitude and phase spectra, extracted as defined in Equations (3) and (4), are stored for the history of two preceding signal segments (at time-instants *m* − 1 and *m* − 2). Based on this, the current signal segment's amplitude, *Â*[*m*, *k*], and phase, *ϕ̂*[*m*, *k*], spectra estimates are calculated, as shown in Equation (7):

(7) $$\begin{array}{cc} \hat{A}\left[m,k\right]=2A\left[m-1,k\right]-A\left[m-2,k\right], & \hat{\phi }\left[m,k\right]=2\phi \left[m-1,k\right]-\phi \left[m-2,k\right] \end{array}$$

The amplitude and phase spectrum estimates are used for the calculation of weight coefficients *W*[*m*, *k*], defined in Equation (8). *W*[*m*, *k*] is proportional to the estimation error of each frequency component, *k*, in the current signal segment, *m*.

(8) Re(X^[m,k])=A^[m,k]cos(ϕ^[m,k])Im(X^[m,k])=A^[m,k]sin(ϕ^[m,k])W[m,k]=(Re(X[m,k])−Re(X^[m,k]))2+(Im(X[m,k])−Im(X^[m,k]))2A[m,k]+|A^[m,k]|

*W*[*m*, *k*] is then used to calculate the weighted segment's energy, *E_w_*[*m*], shown in Equation (9):

(9) $$E_{w}\left[m\right]=\sum_{k}W\left[m,k\right]P\left[m,k\right]$$

Finally, by comparing the weighted and unweighted segment's energy, tonal index *T*[*m*], related to the current signal segment, is defined in Equation (10). Based on this, the temporal feature, *δm_tonal_*, describing the duration of tonal sections, is extracted.

(10) $$T\left[m\right]=log_{2}\frac{E_{w}\left[m\right]}{E\left[m\right]}$$

#### 4.2.3. Power Spectrum Peaks

Within each signal segment, *m*, the potential locations of frequency components originating from wheezing are first identified by searching the segment's power spectrum, *P*[*m*,*k*], for indices at which local maxima (peaks) occur. The peaks' magnitudes, *P_peak_*[*m*,*p*], their total number, *N_p_*[*m*] Equation (11), and their frequencies, *k_peak_*[*m*, *k*] Equation (12), are extracted:

(11) $$\begin{array}{ccc} P_{\text{peak}}\left[m,p\right]=\{P[m,k]: & P[m,k]>P[m,k+1],P[m,k-1]\}, & P=1\ldots N_{p}[m] \end{array}$$

(12) $$k_{\text{peak}}\left[m,p\right]=\left\{k:\;P\left[m,k\right]=P_{\text{peak}}\left[m,p\right]\right\}$$

#### 4.2.4. Entropy of Power Spectrum Peaks

Due to its property of expressing signal complexity, we evaluate Shannon's entropy as a detector of grouping in the spectrum, thus indicating the occurrence of wheezing. We calculate it similarly as proposed in [37].

First, extracted power spectrum peaks *P_peak_*[*m*, *p*] are rescaled according to Equation (13) to produce normalized spectral peaks *P_norm_*_,_*_peak_*[*m*, *p*]:

(13) $$P_{\text{norom},\text{peak}}\left[m,p\right]=\frac{P_{\text{peak}}\left[m,p\right]}{\sum_{p}P_{\text{peak}}\left[m,p\right]}$$

Then, the signal segment's entropy, *En*[*m*], is expressed as in Equation (14):

(14) $$En\left[m\right]=-\sum_{p}\left(P_{\text{norm},\text{pea}k}\left[m,p\right]\cdot log_{2}\left(P_{\text{norm},\text{peak}}\left[m,p\right]\right)\right)$$

The most noticeable changes in entropy are expected upon the transition between signal segments of the normal respiratory sound and segments containing wheezing. Thus, a temporal feature, *En_ratio_*[*m*], defined in Equation (15), describing the ratio of entropies of two successive signal segments, is extracted.

(15) $$En_{\text{ratio}}\left[m\right]=\frac{En\left[m\right]}{En\left[m-1\right]}$$

#### 4.2.5. Spectral Crests Modeled by Low-Order Statistical Moments

**Figure 4.**
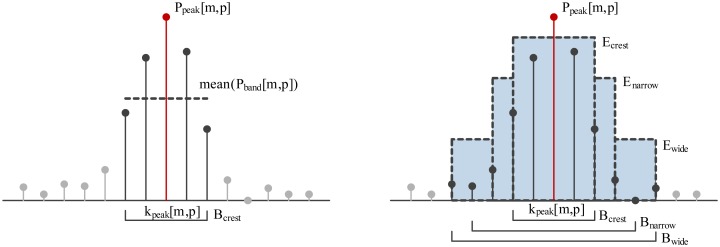
(**Left**) Low-order statistical model of the crest shape; (**Right**) Crest shape modelled by energy.

The first approach to spectral crest modeling is based on the first- and second-order statistical moments (mean, standard deviation) describing the distribution of the magnitudes of the subset of power spectrum components, *P_band_*[*m*, *p*], forming a band around the central frequency, *k_peak_*[*m*, *p*], of the each of the *N_p_* power spectrum peaks (see Equation (16)). Bandwidth *B_crest_* (see Figure 4, left) is chosen during algorithm training.

The mean value and the standard deviation of all power spectrum components within each of *p* bands *P_band_*[ *m*, *p*] are calculated. Those peaks, *P_peak_*[*m*, *p*], the magnitudes of which exceed the condition defined in Equation (17), are declared to be the peaks of the spectral crests, *P_crest_*[*m*, *c*], potentially originating from wheezing. The constants, *C_m_*, *C_s_*, are obtained during the training phase. Crest-peak frequencies *k_crest_*[*m*, *k*] and the number of crests, *N_c_*[*m*, *k*], are also extracted, as shown in Equations (17) and (18):

(16) $$P_{\text{band}}\left[m,p\right]=\left\{P\left[m,k_{\text{peak}}\left[m,p\right]-\frac{B_{\text{crest}}}{2}\right]\ldots P\left[m,k_{\text{peak}}\left[m,p\right]+\frac{B_{\text{crest}}}{2}\right]\right\}$$

(17) $$\begin{array}{c} P_{\text{crest}}\left[m,c\right]=\{P_{\text{peak}}\left[m,p\right]:\;P_{\text{peak}}\left[m,p\right]>C_{\text{mean}}\cdot \text{mean}\left(P_{\text{band}}\left[m,p\right]\right)\\ +C_{\text{std}}\cdot \text{stdev}\left(P_{\text{band}}\left[m,p\right]\right)\},\;c=1\ldots N_{c}\left[m\right] \end{array}$$

(18) $$k_{\text{crest}}\left[m,c\right]=\left\{k:P\left[m,k\right]=P_{\text{crest}}\left[m,c\right]\right\}$$

#### 4.2.6. Spectral Crests Modeled by Energy

An alternative approach to spectral crest modeling is to measure the distribution of energy localized around each identified spectral peak. This model is a modified version of the work presented in [18], with the omission of psychoacoustic auditory modeling.

For each of the identified peaks, *P_peak_*[*m*, *p*], three bands are defined, concentrically spanning around the peak frequency, *k_peak_*[*m*, *p*]: *B_crest_* < *B_narrow_* < *B_wide_* (see Figure 4, right). *B_crest_* is the bandwidth containing the main lattice of a single harmonic represented using a combination of the used signal window (e.g., Hamming) and the time-frequency resolution of STFT. *B_narrow_* and *B_wide_* define the surroundings of each spectral peak and are empirically set to 80 or 120 Hz, respectively. Those spectral peaks for which Equation (19) holds are proclaimed crest peaks *P_crest_*[*m*, *c*]. Band energies *E_narrow_*[*m*, *p*] and *E_wide_*[*m*, *p*] are calculated analogously to *E_crest_*[*m*, *p*]. Constants *C_narrow_* and *C_wide_* are obtained during training.

(19) $$\begin{array}{c} P_{\text{crest}}\left[m,c\right]=\{P_{\text{peak}}\left[m,p\right]:\frac{E_{\text{crest}}\left[m,p\right]}{E_{\text{narrow}}\left[m,p\right]}>C_{\text{narrow}}\;\text{and}\;\frac{E_{\text{crest}}\left[m,p\right]}{E_{\text{wide}}\left[m,p\right]}>C_{\text{wide}}\}\\ E_{\text{crest}}\left[m,p\right]=\overset{k_{\text{peak}}\left[m,p\right]+B_{\text{crest}}/2}{\underset{k_{\text{peak}}\left[m,p\right]-B_{\text{crest}}/2}{\text{∑}}}P\left[m,k\right] \end{array}$$

#### 4.2.7. Temporal Features of Crests

Two temporal features of spectral crests are derived in order to discriminate longer spectral crests originating from wheezing from the short, isolated transients in the time-frequency plane.

**Figure 5.**
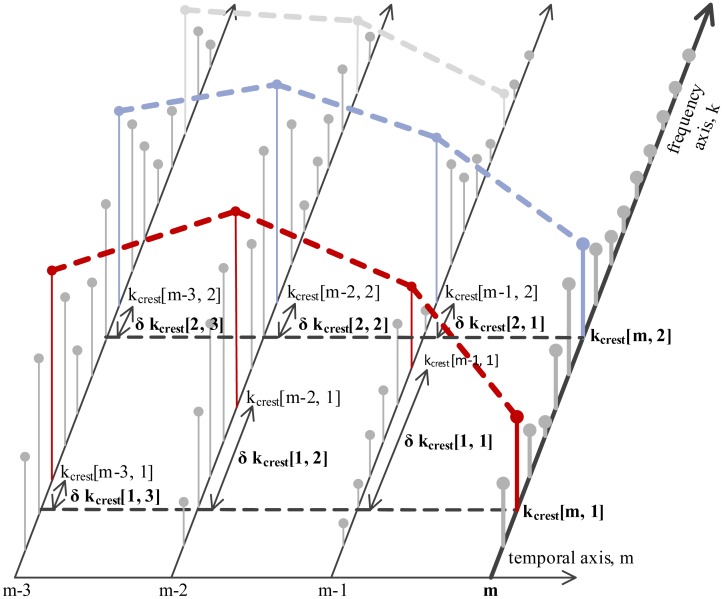
Tracking spectral crests in the temporal plane for continuity and duration.

The first feature is the continuity of the spectral crests in the time-frequency plane. Continuity is described by extracting the deviations of each crest's peak frequency, *k_crest_*[*m*,*c*], along the temporal axis, as shown in Figure 5. Deviations *δk_crest_*[1…*M_cont_*,*c*] are extracted pairwise between the current signal segment, *m*, and each of its *M_cont_* preceding neighbor segments, as shown in Equation (20). The second temporal feature is the duration, *δm_crest_* [*c*], of each continuous spectral crest.

(20) $$\begin{array}{c} \delta k_{\text{crest}}\left[1,c\right]=\left|k_{\text{crest}}\left[m,c\right]-k_{\text{crest}}\left[m-1,c\right]\right|\\ \ldots \\ \delta k_{\text{crest}}\left[M_{\text{cont}},c\right]=\left|k_{\text{crest}}\left[m,c\right]-k_{\text{crest}}\left[m-M_{\text{cont}},c\right]\right| \end{array}$$

The computational complexity of each feature extraction program block is listed in Table 4.

### 4.3. Decision Tree Classification

**Figure 6.**
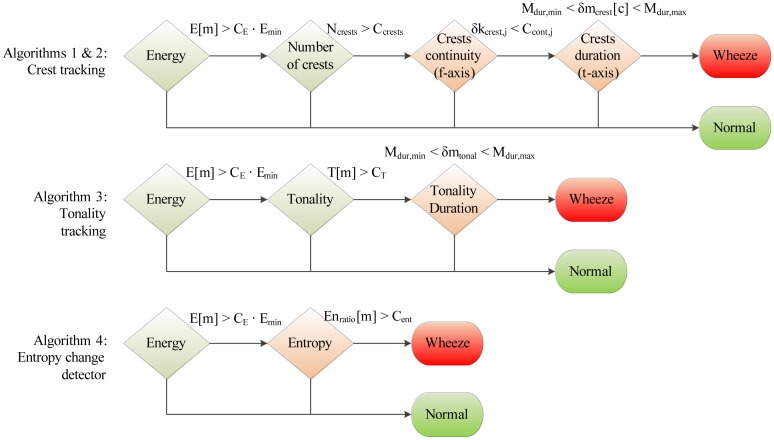
Decision trees of the implemented wheeze detection algorithms.

A total of four wheeze detection algorithms are developed, by organizing subsets of features from Section 4.2 into decision trees, shown in Figure 6: two crest tracking algorithms sharing the analogous decision trees (labeled Algorithms 1 and 2), a tonality tracking algorithm (Algorithm 3) and an entropy change detector (Algorithm 4). All decision trees share the same root, evaluating the segment energy, in order to decide whether the segment is part of a respiratory cycle or an inter-respiratory pause, enabling early termination. The remaining branches are algorithm-specific. The classification operates segment-wise, assigning each signal segment to one of two classes: “non-wheezing”, or “wheezing”.

#### 4.3.1. Algorithms 1 and 2: Crest Tracking

First, the existence of spectral crests is determined by modeling the surroundings of the power spectrum peaks, either using statistical moments as in Algorithm 1 (see Equation (17)), or as in Algorithm 2, using energy (see Equation (19)). Extracted crests are counted in order to check that feature *N_c_* satisfies 1< *N_c_* < *C_crests_*. Next, the temporal features of crests are evaluated.

First, the continuity describing features *δk_crest_*[1, *c*]…*δk_crest_*[*M_cont_*, *c*] are checked against *M_cont_* thresholds *C_cont_*[1]…*C_cont_*[*M_cont_*] according to Equation (21). Those spectral crests satisfying the condition are considered continuous. For *M_cont_* > 1, individual deviation thresholds may be chosen. Constants *C_cont_*[1]… *C_cont_*[*M_cont_*] are acquired during training.

(21) $$\delta k_{\text{crest}}\left[1,c\right]<C_{\text{cont},1}\;\text{and}\;\delta k_{\text{crest}}\left[2,c\right]<C_{\text{cont},2}\ldots \;\text{and}\;\delta k_{\text{crest}}\left[M_{\text{cont}},c\right]<C_{\text{cont},M}$$

Finally, the duration, *δm_crest_*[*c*], of each spectral crest is evaluated to lie between the minimal and maximal durations, *M_dur_*_,_*_min_*, and *M_dur_*_,_*_max_*, respectively. *M_dur_*_,_*_min_* is adjusted to the duration defining continuity, *M_dur_*_,_*_min_* = *M_cont_*. *M_dur_*_,_*_max_* is chosen to reflect the maximal expected uninterrupted duration of wheezing, typically being a duration of the respiratory cycle.

#### 4.3.2. Algorithm 3: Tonality Tracking

The tonality tracking algorithm calculates the tonality of each signal segment according to Equations (7)–(10). Segments satisfying *T*[*m*] > *C_T_* are considered tonal. Constant *C_T_* is acquired through training. In the final decision tree branch, the duration of the successive signal segments marked as tonal, *δm_tonal_*, is compared against constants *M_dur_*_,_*_min_* and *M_dur_*_,_*_max_*.

#### 4.3.3. Algorithm 4: Entropy Change Detection

The algorithm is designed to detect transitions between the interval of normal respiration and the interval containing wheezing. It compares the ratio of entropies, *En_ratio_*[*m*] (see Equations (13)–(15)), against a threshold, *C_ent_*. The threshold is acquired during training.

The total computational complexity estimates of all algorithms are shown in Table 5. They are obtained by summing the complexities of those program blocks from Tables 3 and 4, participating in each algorithm according to Figure 6.

## 5. Experimental Evaluation Methodology

### 5.1. Hardware Platform and Implementation

**Figure 7.**
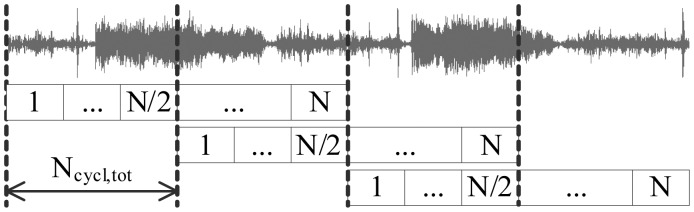
The organization of the processing task on the digital signal processor (DSP).

The algorithms described in Section 4.3 were first implemented in MATLAB and afterwards ported to DSP. A development board EZDSP-C5505-USB (Texas Instruments) [43] was used for prototyping of the wheeze detection sensor node. The board features an analog audio input/output interface, a TLV320AIC3204 analog to digital converter (ADC), a TMS320C5505 DSP core, an universal asynchronous receiver/transmitter (UART) and a debugging interface XDS-1000. The signal was digitized at the ADC's sampling frequency of *f_s_* = 8, 000 Hz. The Inter-integrated circuit sound bus (I2S) was used for the signal transport from the ADC to the DSP. The direct memory access (DMA) units' interrupts were used for the synchronization of the main processing tasks: (a) the signal acquisition task; and (b) the classification task; shown in Figure 7.

The classification task operated on fixed-sized signal segments of *N* = 512 samples, corresponding to 64 ms. The task resulted in declaring each segment to either be the “wheezing” or “normal” class. The result was output by UART. To compensate for the signal attenuation around the cosine window edges, segments were overlapped by 50%, resulting in a total of 32 ms available for the processing of each signal segment. With the DSP core operating at a 100 MHz clock, this sufficed for maximally *N_cycl_*_,_*_tot_* = 3.2 × 10^6^ single-cycle instructions for the processing of each segment, and this yields a power consumption of approximately 22 mW. For the remainder of the cycle, the DSP is kept in standby state, while the DMA periphery performs the acquisition task, while consuming only 0.4 mW. The DSP is woken up upon the DMA's interrupt.

Texas Instruments “DSPlib” library functions [42] were used for the implementation of the common signal processing functions, such as algebraic operations on vectors, trigonometric, logarithmic functions, statistical functions, FFT, *etc*., in 16-bit fixed-point arithmetic. This ensures the reproducibility of the results and optimizes the execution performance by exploiting C5505′s architectural features, such as two multiply-and-accumulate (MAC) units and the FFT coprocessor.

### 5.2. Test Signals

The wheeze detection algorithms were tested on a database of prerecorded respiratory sounds. Our database consisted of a total of 26 recordings. Thirteen of them were of normal breathing (N01…N13), and each of the other 13 audio recordings, labeled W01…W13, contained more than one uninterrupted interval of wheezing. The number of recordings used in our study corresponds to the dataset sizes used throughout the literature (see Table 1, column “Dataset”). Due to the lack of a single standard respiratory sound database, the recordings used in our study were drawn from multiple commonly referenced Internet sources [44–48], and some were recorded in the course of previous research [49].

Table 6 provides the details of each recording. “Dur.” is the duration of the recording in seconds. “Seg.” refers to the number of 50%-overlapped 64-ms signal segments. “Resp. phases” is the total count of inspiratory and expiratory phases. “Seg.” and “Resp. phases” define the number of samples used in the statistical evaluation of results. “Wheeze intervals” are the count numbers of the intervals of wheezing within each recording. “Sample rate” is the frequency at which the recording was originally digitized.

### 5.3. Testing Environment

**Figure 8.**
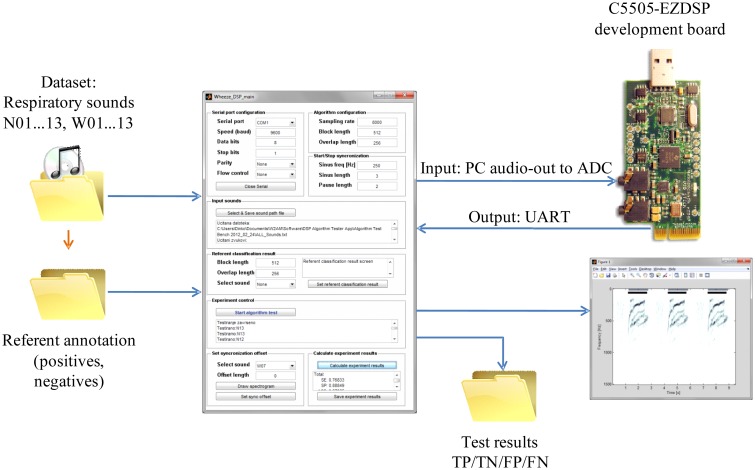
The test environment used for the automated assessment of the classification accuracy of the algorithms running on the DSP. ADC, analog to digital converter.

An environment for signal annotation, algorithm training and testing was designed in MATLAB (see Figure 8). The annotation of the referent classification results was performed by an expert's audio-visual inspection of the signals' waveforms and spectrograms. Intervals containing normal respiratory sounds were annotated as negative (N) and intervals containing wheezing as positive (P). The number of annotated intervals of wheezing is provided for each signal, W01…W13, in column “Wheeze intervals” of Table 6. The temporal resolution of the annotations is adjusted to the segment size upon which the wheeze detection algorithm was running on the DSP (determined by the signal segment size, the overlap and the development board's ADC sampling frequency).

To simulate a realistic signal chain, training and testing was conducted by outputting test signals through the PC sound-card line-out to the C5505-EZDSP development board's ADC input. The results of the segment-wise two-class classification (“normal” or “wheezing”) were returned from the DSP to the PC through UART. Comparing each classification result of each (64 ms) signal segment to the referent annotation, each segment was designated into one of four categories, true positive (TP), true negative (TN), false positive (FP) or false negative (FN), enabling the calculation of the number of classification results belonging to each category (*N_TP_*, *N_TN_*, *N_FP_* and *N_FN_*).

### 5.4. Experiments

#### 5.4.1. Testing of Classification Accuracy

Classification accuracy was tested in two operating scenarios:

1. Wheeze duration tracking scenario. In this scenario, the dataset used for statistical evaluation consisted of a total of 4,422 segments of normal respiratory sounds, N01…N13, and 5,452 segments containing wheezing (W01…W13), each segment corresponding to 64 ms of sound. For details, please refer to column “Seg.” in Table 6. *N_TP_*, *N _FP_*, *N _TN_* and *N _FN_* were calculated segment-wise.
2. Detection of the occurrence of wheezing in a respiratory phase. For this scenario, the annotations of the test-signals were readjusted for the classification results evaluated respiratory phase-wise. Whole respiratory phases containing more than one interval of wheezing were annotated as referent positives, and the phases without the occurrence of wheezing as referent negatives. Thus, the dataset consisted of a total of 65 positives (found throughout W01…W13) and 148 negatives (of those 66 in W01…W13 and the 82 in N01…N13), as seen from column “Resp. phases” in Table 6. *N_TP_*, *N _FP_*, *N _TN_* and *N _FN_* were calculated based on the classification results obtained for each respiratory phase. Due to the DSP still operating segment-wise, the following mapping is introduced: the respiratory phase containing wheezing (annotated positive) was considered TP if containing at least one positively classified signal segment. Furthermore, the respiratory phase was categorized as FP in the case of the existence of positively detected signal segments in the respiratory phase lacking the occurrence of pathology. This is analogously so for TN and FN.

From *N_TP_*, *N _TN_*, *N _FP_* and *N _FN_*, sensitivity *SE*, specificity *SP* and accuracy *AC* were calculated as defined in Equation (22). Sensitivity measures the fraction of correctly classified samples of wheezing (from the subset of test samples composed only of positives). On the other hand, specificity measures the percentage of correctly classified samples of normal respiration (in a signal containing exclusively negatives), while accuracy measures the overall performance.

(22) $$SE=\frac{N_{TP}}{N_{TP}+N_{FN}},SP=\frac{N_{TN}}{N_{TN}+N_{FP}},AC=\frac{N_{TP}+N_{TN}}{N_{TP}+N_{FP}+N_{FN}+N_{TN}}$$

For both operating scenarios, the leave-one-out method was used for training and testing, due to the limited size of the test signal database. The method tested each of *N* = 26 signals from the database, using the classification thresholds obtained through training on the remaining 25 signals. The training of the algorithm thresholds was performed by a grid-search hyper-parameter optimization procedure in which the goal function, shown in Equation (23), was chosen similarly to [15], as the maximum of the area under the curve, *AUC_max_*, of the receiver operating characteristic (ROC), comparing the true positive rate (*TPR* = *SE*) against the false positive rate (*FPR* = 1 − *SP*).

(23) $$AUC_{\text{max}}=\text{max}\left(SE\cdot SP\right)$$

After completing the leave-one-out procedure on all test signals, *SE*, *SP* and *AC* were calculated separately, both for test signals containing intervals of wheezing (W01…W13), for normal signals (N01…N13) and for the whole database, for each of the four algorithms. Training and testing were analogously repeated for both wheeze duration tracking and the wheeze occurrence detection operating scenario, resulting in ${\bar{SE}}_{\text{dur}}$, ${\bar{SP}}_{\text{dur}}$, ${\bar{AC}}_{\text{dur}}$ and ${\bar{SE}}_{\text{event}}$, ${\bar{SP}}_{\text{event}}$, ${\bar{AC}}_{\text{event}}$, respectively.

#### 5.4.2. Execution Duration

Verification of the execution duration was performed using code profiling tools of the Code Composer Studio development environment (Texas Instruments). Algorithms were running on the DSP in debug mode. The time intervals of interest were measured using manually set breakpoints in the number ticks of the DSP core clock running at 100 MHz. A common, representative segment chosen from an interval of wheezing contained in test signal W08 was used throughout all execution duration measurements, yielding the worst case execution time for all algorithms. All measurements were repeated 10 times and averaged.

Using such a setup, the durations of the execution of each program block from Figure 3 were measured. Furthermore, the total time required for the execution of the classification task over the single signal segment, *N̅_cycles_*_,_*_total_*, was measured.

#### 5.4.3. Code Execution Efficiency

In order to evaluate the suitability of the implemented algorithms for long-term wheeze monitoring using a low-power wearable sensor, we assessed their execution efficiency. Therefore, we propose metrics, defined in Equation (24) as *μ_SE_*, *μ_SP_* and *μ_AC_*, comparing, respectively, overall classification sensitivity $\bar{SE}$, specificity $\bar{SP}$ or accuracy $\bar{AC}$ for the processing duty-cycle, *D_exec_*. Processing duty-cycle *D_exec_* is defined as the ratio between the average number of DSP instructions required for the execution of classification task over a single signal segment, *N_cycl_*_,_*_exec_*, and the total number of clock cycles between two successive signal segments (e.g., *N_cycl_*_,_*_tot_* = 3.2 × 10^6^ when the DSP core is running at 100 MHz and the time between successive signal segments equals 32 ms). *D_exec_* is directly related to the portion of time the DSP has to spend in the active state. The efficiency is measured for each of two operating scenarios: wheeze duration tracking (labeled as *μ_SE,dur_*, *μ_SP,dur_* and *μ_AC,dur_*) and wheeze occurrence detection (labeled as *μ_SE,event_*, *μ_SP_*_,_*_event_* and *μ_AC,event_*).

(24) $$\mu_{SE}=\frac{\bar{SE}}{{\bar{D}}_{\text{exec}}},\mu_{SP}=\frac{\bar{SP}}{{\bar{D}}_{\text{exec}}},\mu_{AC}=\frac{\bar{AC}}{{\bar{D}}_{\text{exec}}},\;\text{with}\;{\bar{D}}_{\text{exec}}=\frac{{\bar{N}}_{\text{cycl},\text{exec}}}{{\bar{N}}_{\text{cycl},\text{tot}}}$$

## 6. Results

### 6.1. Accuracy of Classification

**Figure 9.**
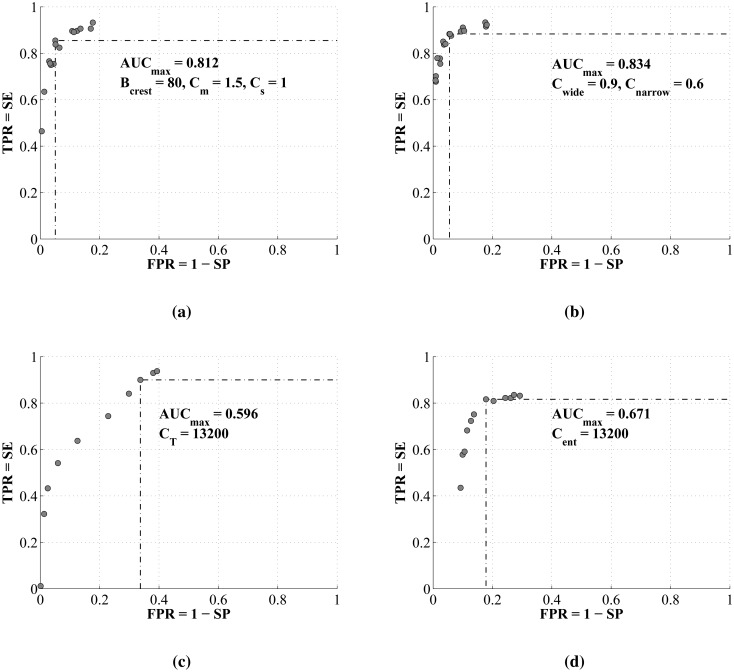
Receiver operating curves of the implemented algorithms. (**a**) Algorithm 1: tracking of crests (stat.moments); (**b**) Algorithm 2: tracking of crests (energy); (**c**) Algorithm 3: tonality tracking; (**d**) Algorithm 4: peak entropy change detection. AUC, area under the curve; TPR, true positive rate; SE, sensitivity; FPR, false positive rate; SP, specificity.

The receiver operating curves averaged through all *N* = 26 iterations of leave-one-out training of each of four algorithms are compared in Figure 9. The maximal areas under the curves, *AUC_max_*, and the associated set of trained classification parameters by which they are obtained, are shown on each graph.

**Figure 10.**
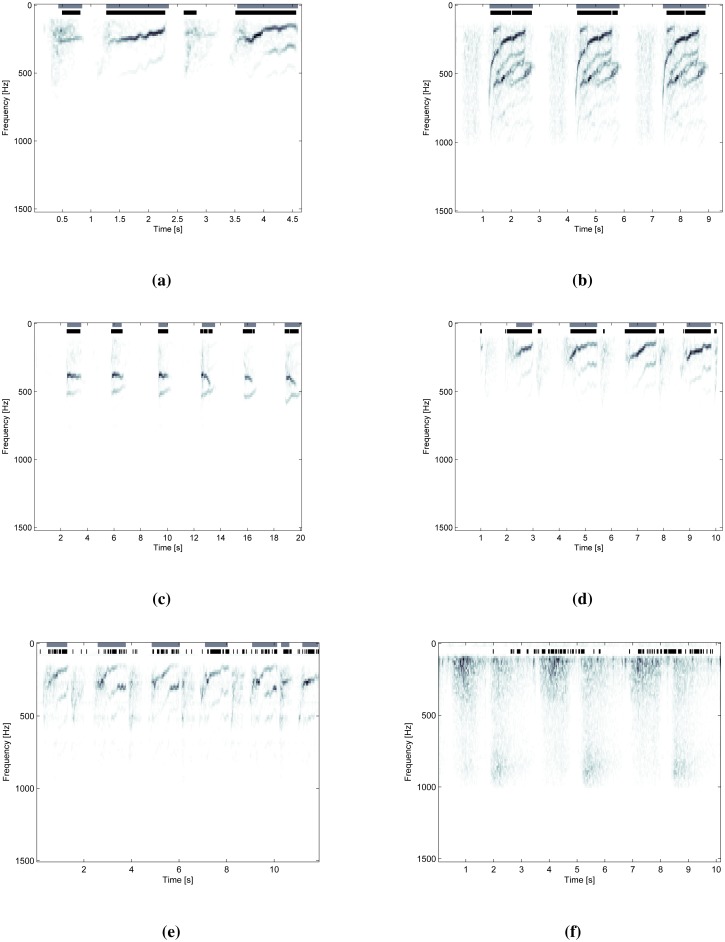
Examples of the classification results (black markings) overlaid onto spectrograms of the test signals and compared to the referent annotation (gray markings). (**a**) Signal W04 classified by Algorithm 1; (**b**) Signal W09 classified by Algorithm 2; (**c**) Signal W07 classified by Algorithm 3; (**d**) Signal W10 classified by Algorithm 4; (**e**) Signal W01 classified by Algorithm 3; (**f**) Signal N03 classified by Algorithm 4.

Figure 10 shows examples of the classification results overlaid onto signal spectrograms. Gray markings represent referent intervals of wheezing annotated by an expert (referent positives), while black markings are signal segments classified as wheezing (classified as positive). Figure 10a–d shows examples of accurate classification by all four algorithms suitable for wheeze duration tracking. On the other hand, Figure 10e shows an example of a less successful classification by Algorithm 3, containing a high number of false negative signal segments. Similarly, Figure 10f shows an example containing a high number of false positive signal segments obtained on normal a respiratory signal by Algorithm 4.

Overall ${\bar{SE}}_{\text{dur}}$, ${\bar{SP}}_{\text{dur}}$, ${\bar{AC}}_{\text{dur}}$, obtained in wheeze duration tracking operating scenario, are shown in Table 7. The values listed in column “Thresholds” refer to the trained threshold values of the classification parameters from Table 8. “W” denotes the results obtained only on W01…W13 and “N” on N01…N13. Event detection accuracies are compared in Table 9, listing only the overall results for brevity. The best results are highlighted in **green**, and the worst are colored **red**.

### 6.2. Execution Duration and Efficiency

**Figure 11.**
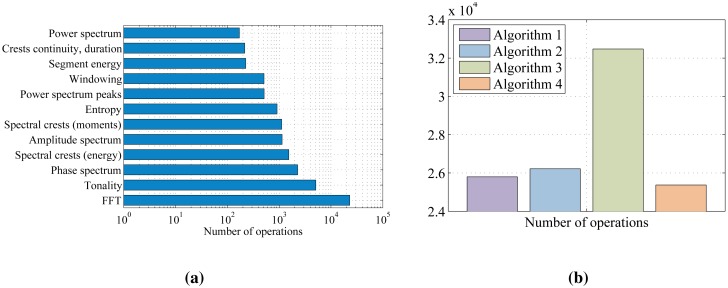
Estimates of the execution durations based on the analysis of *a priori* computational complexities, measured in the number of operations. (**a**) Execution duration estimate of each program block; (**b**) total execution duration estimates.

Execution duration estimates, obtained by calculating expressions from Tables 4 and 5, for a characteristic set of variable values (e.g., *N* = 512, *N_b_* = 57, *N_p_* = 20, *B_c_* = 6, *N_c_* = 7, *M_cont_* = 4, *B_narrow_* = 8, *B_wide_* = 12 and *N_itNR_* = 3, *N_itTA_* = 3, *N_itTA_* = 3, *N_itTC_* = 3, *N_itN_* = 5, *N_itTL_* = 5), are shown in Figure 11. The results are expressed in the number of operations (multiplications and additions) per signal segment. Figure 11a compares the execution duration estimates feature-by-feature, while the total number of operations per each wheeze detection algorithm is given in Figure 11b.

**Figure 12.**
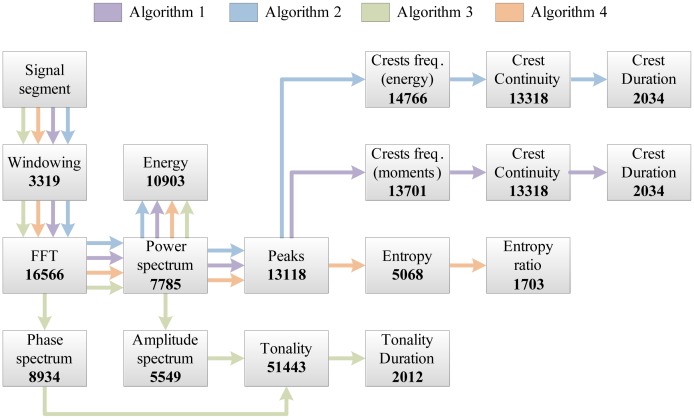
Profiling of the experimentally obtained execution time. Numbers in bold indicate the average number of clock cycles required for each program block.

Figure 12 shows the experimental results of the DSP execution time profiling of each implemented program block, enabling the identification of bottlenecks. Arrows show the execution order and the inclusion of particular program blocks into each of the four implemented algorithms. The values express the average number of DSP cycles required for a single execution of the corresponding program block. The total number of DSP clock cycles required for the worst-case execution of classification task *N̅_clk__,__exec_* and the associated processing duty-cycle based on 32 ms between the processing of successive segments is shown in Table 10. The associated code execution efficiencies are compared in Table 11.

## 7. Discussion

### 7.1. Accuracy of Wheeze Duration Tracking

The receiver operating curves of both crest tracking algorithms (Algorithms 1 and 2) exhibit the highest maximal area under the curve (*AUC_max_*). Additionally, by featuring a clear inflection point, they enable the unambiguous setting of the classification parameter thresholds, which yield the combination of the highest true positive rate (highest sensitivity) at the lowest false positive rate (highest specificity). Good wheeze duration tracking capability can be observed by the examples of the test results in Figure 10a,b and is supported by the highest overall sensitivities, specificities and accuracies. Both algorithms feature, on average, 3%–6% higher specificity than sensitivity (tracking normal signals slightly better than wheezing). Of two versions of the algorithms, Algorithm 2, featuring the energy-based crest model, shows a 1.21% advantage in sensitivity, 3.92% in specificity and 3.52% in accuracy over Algorithm 1, which models spectral crests by low-order statistical moments.

Even though Algorithm 4 (the entropy change detector) also features a receiver operating curve with a clear inflection point, its *AUC_max_* is approximately 15% lower than those of Algorithms 1 and 2. Its maximal sensitivity is limited to 85%, and the specificity converges to less than 90%. Compared to the crest tracking algorithms, Algorithm 4 achieves a lower overall ${\bar{SE}}_{\text{dur}}$, ${\bar{SP}}_{\text{dur}}$ and ${\bar{AC}}_{\text{dur}}$, all equaling around 83% in the wheeze duration tracking scenario.

Algorithm 3 (tonality tracking) features the most shallow receiver operating curve without a clear inflection point. Thus, the algorithm can be adjusted either for high sensitivity at the cost of low specificity (e.g., efficient tracking of wheezing, but a high number of additional false positives in signal segments of normal respiration), or on the other hand, it may be set for high specificity, at the cost of a high count of false negatives during the occurrence of wheezing (a weaker wheeze duration tracking performance, as seen in Figure 10c). When the classification threshold is set in-between, in the ROC's “ramp” region, the results contain a significant amount of both false positives and negatives, keeping the overall accuracy around 70%.

### 7.2. Accuracy of Event Detection

Due to the invariance of the event detection metrics to the occurrence of individual signal segments classified as false negative in the intervals of wheezing (see Section 5.3 and Figure 10e), most successful event detection is expected of those algorithms featuring the receiver operating curves with the highest specificity.

Thus, Algorithms 1 and 2 provide the best overall results in the wheeze-event detection scenario. According to Table 9, Algorithm 1 features the highest sensitivity $\left({\bar{SE}}_{\text{event}}=98.46\%\right)$. Algorithm 2 shows the highest event detection specificity $\left({\bar{SP}}_{\text{event}}=91.21\%\right)$ and accuracy $\left({\bar{AC}}_{\text{event}}=96.92\%\right)$. Generally, crest tracking algorithms feature greater sensitivity than specificity of event detection (better at identifying respiratory cycles containing wheezing). Tonality tracking (Algorithm 3) offers comparable specificity and accuracy to crest tracking algorithms, but lacks sensitivity, meaning that it performs better at identifying respiratory cycles containing only normal breathing. Furthermore, tonality showed 9.4% better accuracy in event detection than the worst performing entropy-based Algorithm 4.

### 7.3. Execution Duration and Efficiency

The results of experimental DSP implementation shown in Table 10 and the *a priori* analysis of the computational complexity shown in Figure 11b agree on the relative relations between the total execution durations of all the algorithms. The differences in the results obtained in the per-feature profiling (Figures 11a and 12) clearly indicate the benefits of the exploitation of DSP's architectural features, which accelerate numerically intensive operations (the FFT coprocessor and dual MAC unit).

According to the experimental results, Algorithm 4 (peak entropy) features the shortest overall execution duration, with the power spectrum peak detection program block being its bottleneck. Algorithms 1 and 2 are slower in execution than Algorithm 4, for 21% and 28%, respectively. They differ only in crest modeling blocks (labeled as “Crest freq.” in Figure 12), with the model based on crest energy being about 7% slower. Tonality tracking tends to be the slowest (a 65% longer execution than Algorithm 4). Its main bottleneck is the numerically intensive calculation of tonality. Furthermore, additional preprocessing blocks (the calculation of the amplitude and phase spectrum) contribute to its total execution time.

According to Table 10, algorithms implemented on the TMS320C5505 DSP range between a 1.87% and 3.09% processing time occupancy (*D̅_exec_*) for a clock set to 100 MHz. Thus, the remaining 96.91%–98.13% of time may be spent in a low power state, minimizing the DSP core consumption. According to Figure 12, on average, 38,573 clock cycles are spent on common signal preprocessing tasks: signal segment windowing, FFT, energy and power spectrum calculation. The rest is algorithm specific.

In spite of its medium classification accuracy, Algorithm 4 (the spectral peaks entropy change detector) turns out to be the most efficient in both operating scenarios, thanks to its very short execution time (see Table 11). In comparison, crest tracking algorithms feature similar execution efficiencies. Compared to Algorithm 4, they are only about 9% lower in the wheeze duration tracking scenario (see *μ_AC,dur_* in Table 11), and *μ_A,event_* is only 5% lower in the event detection scenario. On the other hand, the absolute accuracies of Algorithms 1 and 2 are significantly higher than those of Algorithm 4 (see the associated ${\bar{AC}}_{\text{dur}}$ in Table 7 and ${\bar{AC}}_{\text{event}}$ in Table 9), making them suitable if higher classification accuracy is required. Algorithm 3 (tonality tracking) tends to be the least efficient, about 50% less than Algorithm 4, due to the low accuracy and high execution time.

## 8. Conclusions

In this article, we evaluated the computational complexity, the execution time and the accuracy of the wheeze detection algorithms for optimizing the active time of the DSP of the wearable sensor for real-time asthmatic wheeze detection. Efficiency metrics were introduced comparing the experimentally obtained accuracies and execution durations of four representative algorithms in wheeze occurrence detection and duration tracking scenarios.

The higher classification accuracies of crest tracking algorithms, obtained in both operating scenarios, have shown the advantage over the tonality or entropy-based ones. Though being the least accurate in the wheeze duration tracking scenario, tonality tracking proved more accurate than the entropy-based algorithm and comparable to the tracking of spectral crests modeled using statistical moments, in the event detection scenario.

The implementation of each algorithm required the DSPs activity to be less than 3% of the time, for real-time operation. The highest execution speed was obtained for the entropy-based algorithm and the lowest for tonality tracking (65% lower).

While a general purpose DSP proved valuable for the comparison of different algorithms, it does not define the absolute boundaries of the energy consumption cost of wheeze detection. Nevertheless, such an analysis provides the information necessary for the optimization of the architectural requirements of the DSP unit in future work.

## Figures and Tables

**Table 1.** Summary of the review of wheeze detection algorithms based on STFT signal decomposition. NN, neural network; SVM, support vector machine; VQ, vector quantization; GMM, Gaussian mixture model.

**Year, Author**	**Window, t/f-res.^1^**	**Preprocessing**	**Feature Set**	**Classification**	**Dataset**^2^	**Accuracy**^3^
1992, [32]	cosine, 25.6/39.0	power spectrum, detrend (mean), normalization (stdev)	crest modeling (mean and stdev)	decision tree	not reported	not reported
1995, [33]	rectangular, 100.0/9.8	amplitude spectrum	amplitude spectrum	NN	268 × *W_seg_*, 209 × *N_seg_*	*AC_dur_* = 91.0 − 96.0
1998, [34]	Hann, 42.7/23.5	amplitude spectrogram	t-f continuity of spectral crests (2D gradient)	decision tree	4×*W_subj_*, 4×*N_subj_*, 24–36 s each	*SE_dur_* = 68.0, *SP_dur_* = 70.0
2004, [17]	Hann, 51.2/19.5	power spectrum, detrend (band-wise mean), normalization (stdev)	t-f continuity of spectral crests (mean-based model)	decision tree	16 × *W_subj_*, 15 × *N_subj_*	*SE_n.r._*. = 86.2, *SP_n.r_*. = 96.0
2005, [18]	Hann, 32/15.6	power spectrum	t-f continuity of audible spectral crests (energy based model)	decision tree	4 × *W_subj_*, 12 × *N_subj_*	not reported
2006, [21]	Hann, 11.56/5.4	amplitude spectrogram, detrend (mean)	centroid frequency, duration of closed shapes	decision tree	15 × *W_subj_*, 15 × *N_subj_* (90 × *W_cycle_*, 99 × *N_cycle_*)	*SE_event_* = 96.7, *SP_event_* = 90.9
2007, [20]	cosine, 8.2/19.5	detrend (mean), normalization (stdev), Wavelet denoising	t-f continuity of spectral crests (modeled by mean and stdev)	decision tree	*7* × *W_subj_* (65 × *W_intv_*)	*SE_event_* = 95.4
2007, [19]	Hann, 23.2/2.7	zero padding, detrend by moving average	t-f continuity of spectral crests (mean-based model)	decision tree	13×*W_subj_* (337 × *W_intv_*)	*SE_event_* = 95.5±4.8, *SP_event_* = 93.0 ± 9.3
2008, [35]	not reported	amplitude spectrum	cross-correlation index	empirical threshold	6 × *Wsubj*, *7* × *Nsubj*	*SE_n.r_*. = 83.0, *SP_n.r_*. = 100.0
2008, [36]	Gaussian, 11.6/86.1	power spectrum	mean distortion between histograms of sample entropy	empirical threshold	7 × *W_subj_*, 7 × *N_subj_* (86 × *W_phase_*, 100 ×*N_phase_*)	*SE_n.r_*. = 83.0, *SP_n.r_*. = 100.0
2009, [37]	Hann, 60/15.6	normalized power spectrum, maxima	change of Shannon entropy (ratio, difference)	empirical threshold	10 × *W_subj_*, 7 × *N_subj_*	*AC_n.r_*. = 84.4
2009, [38]	Hamming, 26/37.5	amplitude spectrum	*f*_50_/*f*_90_ + time-domain zero-crossings, kurtosis and Renyi entropy	FDA, Neyman Paersons	7×*W_subj_* (246 × *W_seg_*, 246 × *N_seg_*)	*AC_dur_* = 93.5
2009, [22]	45.5/8.8	spectrogram, Laplacian 2D filtering, half-thresholding	spectrogram projection	NN	40 × *W_cycle_*,72 × *N_cycle_*)	*SE_event_* = 86.1, *SP_event_* = 82.5, *AC_event_* = 84.3
2009, [15]	170.6/5.9	power spectrum	26 power spectrum sub-bands	NN, VQ, GMM	12 × *W_subj_*, 12 × *N_subj_*	*SE_event_* = 87.0, *SP_event_* = 85.0
2011, [23]	Gaussian, not reported	spectrogram, spectral dominance	continuity, position, spread, sparseness	SVM	14 × *W_subj_*, 7 × *N_subj_* (305×*W_phase_*, 284 × *N_phase_*)	*AC_event_* = 92.7±2.9
2011, [39]	cosine, 128/7.8	feature-dependent	kurtosis, *f*_50_/*f*_90_, Shannon's entropy, spectral flatness, tonality index	SVM	28 × *W_subj_*, 28 × *N_subj_*	*AC_dur_* = 85.0 − 92.0

1. Temporal resolution is reported in milliseconds. Frequency resolution is reported in Hertz/bin.

2. *W* denotes part of the dataset with sounds of “wheezing” (containing positives). *N* corresponds to “non-wheezing” (only negatives). Subscripts denote the method of reporting the dataset size: *subj*, number of subjects (patients); *cycle*, number of respiratory cycles; *phase*, number of respiratory phases; *intv*, number of signal intervals (e.g., uninterrupted intervals of wheezing); *seg*, signal segment of the fast Fourier transform's (FFT) window-length.

3. Accuracy is reported using standard statistical metrics, in (%): *SE*, sensitivity; *SP*, specificity; *AC*, accuracy. For definitions, see Section 5.4. Subscripts define the fidelity upon which the accuracy was calculated: *dur*, duration of wheezing was measured; *event*, occurrences of sequences of wheezing are counted; *n.r*., not reported.

**Table 2.** The computational complexity of elementary mathematical functions.

**Function**	**Implementation**	**Multiplications**	**Additions**
*x*/*y*	Newton–Raphson method, in *N_itNR_* iterations	3*N_itNR_*	*N_itNR_*
$\sqrt{x}$	Newton's algorithm, in *N_itN_* iterations	2*N_itN_*	2 *N_itN_*
*log*_2_(*x*)	Taylor series, in *N_itTL_* terms	*N_itTL_*(*N_itTL_* + 1)/2	*N_itTL_*
*sin*(*x*)	Taylor series, in *N_itTS_* terms	(2*N_itTS_* − 1)^2^	*N_itTS_*
*cos*(*x*)	Taylor series, in *N_itTC_* terms	(2*N_itTC_*−2)(2*N_itTC_* −1)	*N_itTC_*
*arctg*(*x*)	Taylor series, in *N_itTA_* terms	(2*N_itTA_* − 1)^2^	*N_itTA_*

**Table 3.** The computational complexity of the signal decomposition program blocks.

**Program Block**	**Comment**	**Multiplications**	**Additions**
Windowing and STFT, Equation (1)	calculated for the signal segment of length *N* by radix-2 decimate-in-time FFT	2*N* log_2_(*N*)	3*N* log_2_(*N*)
Power spectrum, Equation (2)	calculated for *N_b_* < *N* bins corresponding to the bandwidth of respiration	2*N_b_*	*N_b_*
Amplitude spectrum, Equation (3)	calculated on *N_b_* bins, the square root is implemented as in Table 2	2*N_b_ N_itN_*	2*N_b_ N_itN_*
Phase spectrum, Equation (4)	calculated on *N_b_* bins, division and arctg implemented as in Table 2	*N_b_*((2*N_itTA_* −1)^2^ + 3*N_itNR_* + 1)	*N_b_*(*N_itNR_* + *N_itTA_* − 1)

**Table 4.** The computational complexity of feature extraction program blocks, with variables defined in Table 2.

**Feature**	**Multiplications**	**Additions**
Segment energy, Equations (5)–(6)	*N_b_*	3*N_b_*
Tonality, Equations (7)–(10)	*N_b_*((2*N_itTC_* − 2)(2*N_itTC_* − 1) + (2*N_itTA_* − 1)^2^ + *t*2*N_itN_* +3*N_itNR_* +5)+*N_itTL_*(*N_itTL_* + 1)/2	*N_b_*(*N_itTC_* + *N_itTA_* + 2*N_itN_* + *N_itNR_* +1)+*N_itNR_* +*N_itTL_*
Power spectrum peaks, Equation (11)	-	$2N_{b}+N_{p}^{2}$
Entropy, Equations (13)–(15)	*N_p_*(*N_itTL_*(*N_itTL_* +1)+ 3)+3*N_itNR_*	*N_p_*(2(*N_itTL_* − 1) + *N_itNR_* + 1) +*N_itNR_*
Spectral crests (moments), Equation (17)	*N_p_*(3*B_crest_* +2*N_itN_* + 2)	*N_p_*(3*B_crest_* +2*N_itN_* −2)
Spectral crests (energy), Equation (19)	*N_p_*(*B_crest_* +*B_narrow_* +*B_wide_* + 2(3*N_itNR_* + 1))	*N_p_*(*B_crest_* + *B_narrow_* + *B_wide_* + 2(*N_itNR_* + 1) − 3)
Crests continuity and duration, Equation (20)	-	$M_{\text{cont}}N_{c}^{2}+3N_{c}$

**Table 5.** The total computational complexity of each implemented algorithm. For definitions of the variables, see Table 2.

**Algorithm**	**Multiplications**	**Additions**
Algorithm 1	*N*(2*log*_2_*N* + 1) + 3*N_b_* + *N_p_*(3*B_crest_* + 2*N_itN_* + 2)	$3N\log_{2}N+6N_{b}+N_{p}^{2}+N_{p}\left(3B_{\text{crest}}+2N_{\text{itN}}-2\right)+N_{c}\left(M_{\text{cont}}+1\right)$
Algorithm 2	*N*(2*log*_2_*N* + 1) +3*N_b_* + *N_p_*(*B_crest_* +*B_narrow_*+ *B_wide_* +6*N_itNR_* + 2)	$3N\log_{2}N+6N_{b}+N_{p}^{2}+N_{p}\left(B_{\text{crest}}+B_{\text{narrow}}+B_{\text{wide}}+2\left(N_{\text{itNR}}+1\right)-3\right)+N_{c}\left(M_{\text{cont}}+1\right)$
Algorithm 3	*N*(2*log*_2_*N* +1)+*N_b_*(2(2*N_itTA_* −1)^2^ +(2*N_itTC_* −2)(2*N_itTC_* − 1) + 4*N_itN_* + 6*N_itNR_* + 9) + *N_itTL_*(*N_itTL_* + 1)/2	3*Nlog*_2_*N* +*N_b_*(4*N_itN_* +*N_itTC_* +*N_itTA_*+ *N_itNR_* +5)+*N_itNR_* +*N_itTL_*
Algorithm 4	*N*(2*log*_2_*N* + 1) + 3*N_b_* + *N_p_*(*N_itT L_*(*N_itT L_* + 1) + 3) + 3*N_itNR_*	$3N\log_{2}(N)+6N_{b}+N_{p}^{2}+N_{p}(2(N_{\text{itTL}}-1)+N_{\text{itNR}}+1)+N_{\text{itNR}}$

**Table 6.** Database of respiratory signals. “Dur.” is the duration of the recording in seconds. “Seg.” refers to the number of 50%-overlapped 64-ms signal segments. “Resp. phases” is the total count of inspiratory and expiratory phases. “Seg.” and “Resp. phases” define the number of samples used in the statistical evaluation of results. “Wheeze intervals” are the count numbers of the intervals of wheezing within each recording. “Sample rate” is the frequency at which the recording was originally digitized. In the labels column, N stands for normal breathing, while W stands for wheezing.

**Normal Respiratory Sounds**					**Pathological Respiratory Sounds**					
	
**Label**	**Dur.(s)**	**Seg.**	**Resp. Phases**	**Sample Rate (kHz)**	**Label**	**Dur.(s)**	**Seg.**	**Resp. Phases**	**Wheeze Intervals**	**Sample Rate (kHz)**
N01	12.76	398	8	44.1	W01	11.89	371	11	7	44.1
N02	13.48	421	8	44.1	W02	26.73	835	10	5	22.05
N03	10.09	315	6	11.025	W03	15.83	494	16	7	22.05
N04	6.48	202	4	11.025	W04	4.60	143	4	3	11.025
N05	3.12	97	2	11.025	W05	8.04	251	7	3	11.025
N06	6.32	197	4	11.025	W06	10.10	315	9	4	11.025
N07	5.33	166	4	11.025	W07	20.00	625	12	6	8
N08	5.34	167	4	11.025	W08	7.52	235	6	3	11.025
N09	12.86	402	6	8	W09	9.48	296	6	3	44.1
N10	10.09	315	11	11.025	W10	10.15	317	8	4	8
N11	20.00	625	10	8	W11	29.96	936	30	15	44.1
N12	9.99	312	4	11.025	W12	10.60	331	6	2	8
N13	25.76	805	11	44.1	W13	9.70	303	6	3	8

Total	141.62	**4,422**	**82**		Total	174.60	**5,452**	**131**	65	

**Table 7.** Comparison of wheeze duration tracking accuracy. Threshold values relate to the parameters from Table 8 and were trained according to the procedure described in Section 5.4.

**Algorithm**	**Thresholds**	**${\bar{SE}}_{\text{dur}}(\%)$**			**${\bar{SP}}_{\text{dur}}(\%)$**			**${\bar{AC}}_{\text{dur}}(\%)$**		
		
		**W**	**N**	**overall**	**W**	**N**	**overall**	**W**	**N**	**overall**
Algorithm 1	80, 1.5, 1.0	84.41	-	86.30	91.19	88.24	89.50	91.32	88.24	89.01
Algorithm 2	0.9, 1.6	85.90	-	87.51	92.71	94.36	93.42	92.42	94.36	92.53
Algorithm 3	27500	61.36	-	80.10	100	99.76	70.56	70.36	99.76	71.98
Algorithm 4	12600	78.69	-	82.54	80.89	84.59	83.64	82.30	80.89	83.79

**Table 8.** Definitions of the training parameters.

**Algorithm**	**Parameters**	**Description**	**Equation**
Algorithm 1: crests (moments)	*B_crest_*, *C_m_*, *C_s_*	crest width, mean, standard deviation	Equations (16) and (17)
Algorithm 2: crests (energy)	*C_wide_*, *C_narrow_*	crest/band energy ratios: wide; narrow	Equation (19)
Algorithm 3: tonality tracking	*C_T_*	segment tonality threshold	Equation (10)
Algorithm 4: entropy change	*C_ent_*	entropy ratio threshold	Equation (15)

**Table 9.** Comparison of the overall event detection accuracy.

**Algorithm**	**Thresholds**	**${\bar{SE}}_{\text{event}}(\%)$**	**${\bar{SP}}_{\text{event}}(\%)$**	**${\bar{AC}}_{\text{event}}(\%)$**
Algorithm 1	100, 3.0, 1.5	98.46	81.08	86.39
Algorithm 2	1.2, 1.5	96.92	91.21	92.96
Algorithm 3	13,200	76.92	89.86	85.92
Algorithm 4	10,000	87.69	73.28	76.52

**Table 10.** Average experimentally obtained execution durations and processing duty-cycle.

**Algorithm**	*N̅_clk_*_,_*_exec_*	*D̅_exec_* (%)
Algorithm 1	77,287	2.42
Algorithm 2	82,888	2.59
Algorithm 3	98957	3.09
Algorithm 4	59998	1.87

**Table 11.** Execution efficiencies in wheeze duration tracking and event detection scenarios.

**Algorithm**	*μ_SE_*_,_*_dur_*	*μ_SP_*_,_*_dur_*	*μ_AC_*_,_*_dur_*	*μ_SE_*_,_*_event_*	*μ_SP_*_,_*_event_*	*μ_AC_*_,_*_event_*
Algorithm 1	35.66	36.98	36.78	40.68	33.50	35.70
Algorithm 2	33.78	36.07	35.72	37.42	35.22	35.89
Algorithm 3	25.92	22.83	23.29	24.89	29.08	27.81
Algorithm 4	44.14	44.73	44.81	46.89	39.19	40.92
